# The aspiration test reveals an instability of the posterior horn of the lateral meniscus in almost one-third of ACL-injured patients

**DOI:** 10.1007/s00167-021-06806-2

**Published:** 2021-11-27

**Authors:** Christophe Jacquet, Caroline Mouton, Amanda Magosch, George A. Komnos, Jacques Menetrey, Matthieu Ollivier, Romain Seil

**Affiliations:** 1 Sports Clinic, Centre Hospitalier de Luxembourg, Clinique d’Eich, 78, rue d’ Eich, 1460 Luxembourg, Luxembourg; 2 Department of Orthopedic Surgery and Traumatology, Institute for Movement and Locomotion (IML), St. Marguerite Hospital, Marseille, France; 3 Luxembourg Institute of Research in Orthopaedics, Sports Medicine and Science, Luxembourg, Luxembourg; 4 Human Motion, Orthopaedics, Sports Medicine and Digital Methods, Luxembourg Institute of Health, Luxembourg, Luxembourg; 5 Centre de Medecine du Sport et de l’Exercice, Swiss Olympic Medical Center, Hirslanden Clinique la Colline, Geneva, Switzerland; 6 Service de Chirurgie Orthopédique, University Hospital of Geneva, Geneva, Switzerland

**Keywords:** ACL, Lateral root tear, Hypermobile meniscus, Instability, Aspiration test, Probing test

## Abstract

**Purpose:**

Anterior cruciate ligament (ACL) injuries often lead to associated injuries of the posterior horn of the lateral meniscus (PHLM). Arthroscopic, assessment of PHLM instability may be difficult in the absence of a visible meniscus damage. The main objective of this prospective multi-center study was to compare the ability of the probing and aspiration tests to identify PHLM instability in a population of patients undergoing ACL reconstruction (ACLR) and a control group of patients with an intact ACL undergoing knee arthroscopy.

**Methods:**

A prospective case–control analysis was performed in three sports medicine centers. One-hundred and three consecutive patients operated for a primary isolated ACLR without structural lateral meniscus damage other than a root tear were included. They were compared to a control group of 29 consecutive patients who had a knee arthroscopy with an intact ACL and no structural lateral meniscus lesion. The probing and aspiration tests were consecutively executed according to previously published methods.

**Results:**

In the control group, no lateral meniscus lesions were visualized during arthroscopy, and both probing and aspiration tests were negative in all patients. In the group of ACL-injured patients, a Forkel type I–III posterolateral meniscus root tear (PLMRT) was found in 12 patients (12%). In this subgroup, the probing test was positive in 4/12 patients (33%) and the aspiration test in 5 additional patients (75%). In 15 patients (15%), an elongation of the posterior root of the lateral meniscus (defined as type IV PLMRT as an addendum to the Forkel classification) could be observed during arthroscopy. In this subgroup, only 1 patient displayed a PHLM instability with the probing test (7%), whereas the aspiration test was positive in 13/15 patients (87%). In the remaining 76 patients (74%), no structural lesion of the PHLM could be identified. Nevertheless, an instability of the PHLM could be identified in 8 of them (11%) with the probing test, and the aspiration test was positive in 2 additional knees (13%) of this apparently normal subgroup. Altogether, in the entire ACL injury cohort, a positive probing test was observed in 13/103 patients (13%) and a positive aspiration test in 32/103 knees (31%) (*p* < 0.01).

**Conclusion:**

Careful observation and examination of the PHLM with the aspiration test revealed a substantial amount of previously undiagnosed lateral meniscus instabilities in ACL-injured knees. The prevalence of PHLM instability as evaluated by the aspiration test was high (31%). The aspiration test was superior to the probing test in detecting an instability of the PHLM in a population of ACL-injured patients.

**Level of evidence:**

II.

## Introduction

The anatomy of the lateral meniscus is more complex than on the medial side [11]. This is due to complex posterior suspensory structures including the popliteomeniscal fascicles (PMF) and the posterior meniscotibial ligament (MTL) [2, 7, 19], as well as the posterior root and the meniscofemoral ligament which is made of a meniscotibial and a meniscofemoral fixation [3]. Damage to one or several of these structures can result in an instability of the posterior horn of the lateral meniscus (PHLM) which may occur either in isolation or in association with an anterior cruciate ligament (ACL) injury. Whereas the latter may cause a lack of rotational control of the knee, the former usually leads to lateral knee pain or locking sensations [5, 14, 15, 20, 22, 23].

The subluxation occurring in the lateral tibiofemoral compartment during the ACL injury mechanism may cause injuries to the area of the PHLM [12, 16]. Previous publications describing PHLM root tears estimated their prevalence to around 15% [6, 16]. Other injuries to the PHLM without arthroscopically visible structural damage such as posttraumatic instability have been described recently [10], but their prevalence is currently unknown. Since the structures of the PHLM play a role in rotational knee stability [8, 14, 20, 22], it seems crucial to further investigate this new entity. While major structural meniscus damage is easy to identify on MRI or during arthroscopy, assessment of PHLM instability may be difficult due to the lack of reliable clinical tests and imaging signs [21]. Therefore, PHLM instability may currently be easily overlooked. Direct visual dynamic inspection via arthroscopy remains the gold standard. Shin et al. [19] defined a PHLM instability as a displacement of more than half of the lateral meniscus underneath the ‘equator' of the lateral femoral condyle during arthroscopic probing. In cases of limited joint line opening, probing as well as the precise quantification of the amount of subluxation can be difficult. This method is therefore not always reliable to diagnose PHLM instability. Recently, a new arthroscopic test called “the aspiration test” [10] has been proposed as an alternative to the probing test to improve the diagnosis of PHLM instability, but to date, no data are available to confirm this hypothesis.

Therefore, the main objective of this prospective multi-center study was to compare the ability of the probing and aspiration tests to identify PHLM instability between patients undergoing ACL reconstruction (ACLR) and a control group of patients with an intact ACL who underwent knee arthroscopy for other reasons. The hypotheses were that PHLM instability was frequently associated with ACL injuries and underestimated with current arthroscopic methods.

## Materials and methods

A prospective case–control analysis was performed in three sports medicine centers (France, Luxembourg, and Switzerland) between December 2020 and May 2021. The study was performed in accordance with ethical standards. All data were gathered anonymously by the team of clinicians who took care of the patients, so that it did not require prior approval according to our respective law and national ethical guidelines.

The first group of patients (ACL group) consisted of consecutive patients between 18 and 50 years of age who were operated for a primary ACLR. Patients were excluded if they had structural lesions (bucket-handle, vertical, horizontal, or radial tears) of the lateral meniscus other than a posterolateral meniscus root tear (PLMRT) according to the Forkel et al.’s classification [6] as observed during arthroscopic exploration, ACL agenesis, previous knee surgery in the ACL-deficient knee, concomitant collateral (> MRI grade 2) or posterior cruciate ligament injury, associated bone or cartilage procedure, and lateral discoid meniscus. Finally, 103 patients met inclusion and exclusion criteria, and were included.

The control group consisted of consecutive patients between 18 and 50 years of age who underwent knee arthroscopy with an intact ACL as confirmed by MRI. Control patients did not have clinical symptoms of the lateral tibiofemoral compartment and more specifically no clinical suspicion of the rare condition of an isolated PHLM instability. Exclusion criteria included structural lesions (bucket-handle, vertical, horizontal, or radial tears) of the lateral meniscus as observed during arthroscopic exploration, lateral femoral or tibial chondral defects ICRS > 1 [4], previous knee surgery, and lateral discoid meniscus. Finally, 29 patients met inclusion and exclusion criteria, and were included in this group. Indications for knee arthroscopy were collected and distributed as follows: 15 medial meniscus repairs (52%), 6 medial meniscectomies (21%), 5 isolated medial tibiofemoral or patellofemoral chondral defects (17%), and 3 diagnostic arthroscopies (10%).

### Arthroscopic evaluation

**Fig. 1:**
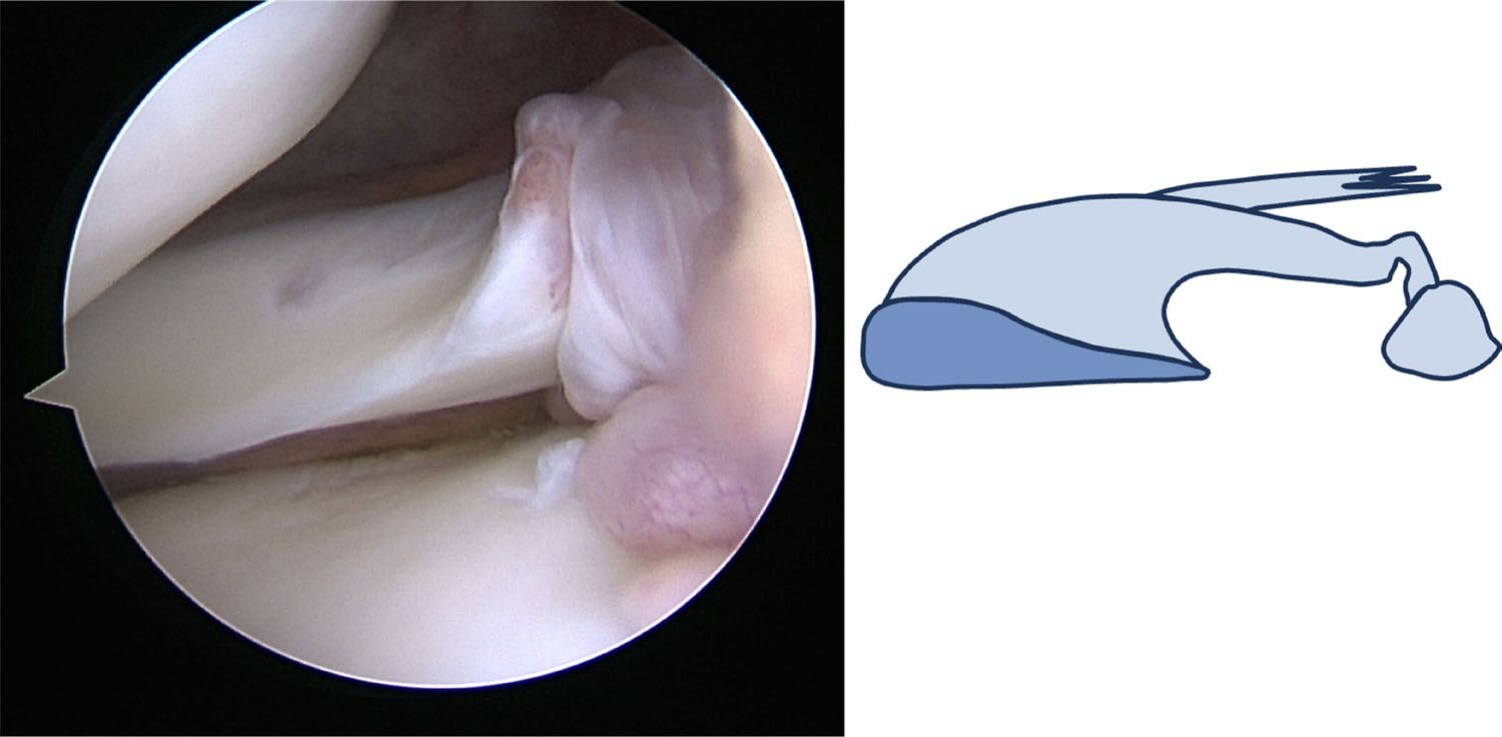
Arthroscopic view and representation of the new type IV posterolateral meniscus root tear

All arthroscopies were performed by three senior surgeons (one in each center). For both the ACL and the control group, a systematic arthroscopic exploration was performed at the beginning of the procedure to confirm the diagnosis and to identify the inclusion and exclusion criteria. The standardized surgical sequence was as follows:

1. Direct visualization of the lateral tibiofemoral compartment
  A classic anterior view with an antero-medial portal with the knee held in a figure-of-4 position was performed. It allowed direct visualization of the chondral status for both the lateral tibial plateau and the lateral femoral condyle using the ICRS classification [4], the lateral meniscus, its posterior root, and the meniscofemoral ligaments. The “lateral gutter drive-through” sign [18] which theoretically allows for the visualization of the posterior tibial plateau, the meniscotibial capsular attachments, and the PMF was not routinely performed in this study. In case of a PLMRT associated or not with a meniscofemoral ligament tear, the Forkel classification [6] was used to categorize the tear. A new type IV lesion called “elongation” was added to this classification (Fig. 1). This type of lesion corresponds to an elongation of the root occurring at the time of the trauma without an identifiable disruption of the meniscotibial fibers, but with fibers that were distended after injury.
2. Probing test
  The arthroscope was placed in the same antero-medial portal with the knee held in a figure-of-4 position. A new antero-lateral portal was created to introduce the probe in the lateral tibiofemoral compartment to reach the PHLM. According to Shin et al. [19], a translation of the lateral meniscus by more than 50% or ‘beyond the equator’ of the lateral femoral condyle was considered as a positive probing test.
3. Aspiration test
  The test was performed as previously described [10]. With the knee held in the figure-of-4 position and flexed to slightly more than 90°, the arthroscope was placed in the antero-lateral or antero-medial portal and directed towards the lateral tibiofemoral compartment. The aspiration test was performed by activating the aspiration of a 4.5 mm shaver (arthroscopy pump: DualWawe, Arthrex, Naples, FL, USA, with standard knee configuration, aspiration shaver: 300 ml/min), and placed at the center of the lateral tibiofemoral compartment. As for the probing test, a translation of the lateral meniscus by more than 50% or ‘beyond the equator’ of the lateral femoral condyle was considered as a positive aspiration test.

### Statistical analysis

The analyses were performed using SPSS software v.25 for Windows.

The Mc Nemar test was used to compare the result (positive or negative) of the probing and the aspirations tests and the kappa coefficient (Kappa Test for Agreement) was reported to indicate the extent of agreement between both tests. Chi-square tests with Bonferroni correction were used to determine whether positive aspiration test, respectively, and positive probing test were associated with the status of the lateral meniscus (no visualized tear, type I–III root tear, and type IV root tear) under arthroscopy. Each expected cell count was checked to ensure that it was greater than five. If more than 20% of cells had an expected cell count inferior to five, the *p* value of likelihood-ratio Chi-square test was considered. To estimate the effect size, Cramer’s *V* was used [15]. Significance was set at *p* < 0.05 for all analyses.

A priori sample size was calculated to obtain a statistical power of 80% and an alpha value of 5% using unpublished pilot data on the probing and aspiration test results of 60 patients. Based on the proportion of negative probing test/positive aspiration test (no event–event) of 10% and the proportion of positive probing test/negative aspiration test (event–no event) of 1%, a minimum sample size of 104 patients was required.

## Results

The status of the lateral meniscus under arthroscopy is presented in Table 1, both for the control and the ACL groups. In the control group the probing and aspiration tests were negative and the lateral meniscus appeared normal under arthroscopy in all patients. In the group of ACL-injured patients, the probing and aspiration tests were positive in 13% (*n* = 13/103) and 31% (*n* = 32/103) of the cases, respectively (Table 2). The difference between the probing and the aspiration test was significant (*p* < 0.01). Both tests showed agreement in 111 out of 132 patients (Kappa agreement = 0.46). In 20 ACL-injured patients (17% of all studied cases), the probing test was negative, while the aspiration was positive. The reverse was true in only one ACL-injured patient.

**Table 1:** Lateral meniscus status in both the ACL and the control group

Type of lateral meniscus tears	ACL group (*n* = 103)	Control group (*n* = 29)
No visible tear	76 (74%)	0
Root tear type I	3 (3%)	0
Root tear type II	2 (2%)	0
Root tear type III	7 (7%)	0
Root tear type IV	15 (15%)	0

**Table 2:** Results of the probing and the aspiration tests in both the ACL and the control group

Group	Type of lateral meniscus tear	*n*	Aspiration test		Probing test	
			Positive	Negative	Positive	Negative
ACL		103	32 (31%)	71	13 (13%)	90
	Root tear types I–III	12	9 (75%)	3	4 (33%)	8
	Root tear type IV	15	13 (87%)	2	1 (7%)	14
	No visible tear	76	10 (13%)	66	8 (11%)	68
Control		29	0	0	0	0
	No visible tear	29	0	0	0	0

**Fig. 2:**
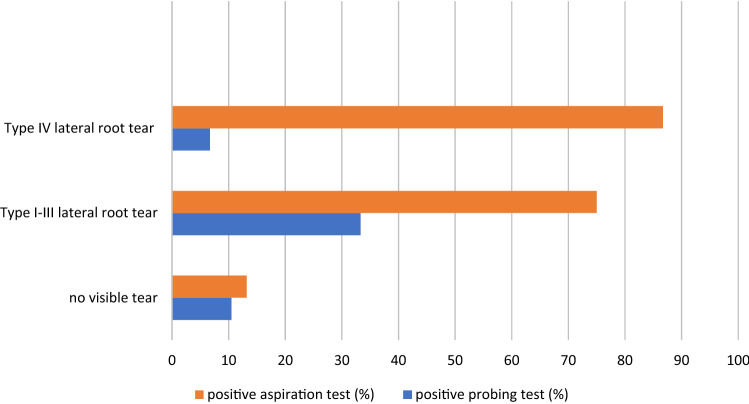
Distribution of positive probing and aspiration tests according to the status of the lateral meniscus in the ACL group

The percentage of positive aspiration tests varied according to the status of the lateral meniscus (no visible tear, type I–III root tear, type IV root tear; *p* < 0.01; Cramer’s *V* = 0.65). The same association could not be confirmed with a positive probing test (n.s). In the presence of a type I–III root tear, the probing test was positive in 4/12 patients (33%) and the aspiration test in 9/12 patients (75%; Kappa agreement between both tests = 0.29). In the presence of a type IV root tear, the probing test was positive in 1/15 patients (7%) and the aspiration test in 13/15 patients (87%; Kappa agreement between both tests = 0.02). In the absence of a visible tear, the probing test was positive in 8/76 patients (10.5%) and the aspiration test in 10/76 patients (13%; Kappa agreement = 0.748) *(Fig. **2**)*.

## Discussion

The main finding of this study was that the aspiration test allowed to identify a prevalence of PHLM instabilities in 31% of ACL-injured patients versus 0 in the control group. Of the 32 patients with a positive aspiration test, 9 had an easily identifiable I–III tear of the PLMR and 13 displayed the newly presented elongation of the PLMR (named type IV as an addendum to the Forkel classification). Finally, 10 ACL-injured patients with a positive aspiration test displayed a PHLM instability despite an arthroscopically intact lateral meniscus. In comparison to the probing test, the aspiration test allowed to better identify instabilities of the PHLM. The superiority of the aspiration test became especially obvious for type IV PLMRT, where more than 85% of the cases could be detected (versus less than 10% with the probing test). The hypothesis that the prevalence of PHLM instability was high in ACL-injured patients (31%) and that PHLM instability was underestimated by the probing test is thus confirmed.

In a recent study, Jacquet et al. [10] described the aspiration test, a method supposed to allow for a better discrimination of PHLM instability as compared to the probing test. The latter was until today the gold standard procedure during routine arthroscopic surgery to identify this condition. According to Shin et al. [19], a translation of the lateral meniscus during the probing test by more than 50% or 'beyond the equator' of the lateral femoral condyle is considered to reflect an instability. However, probing may not necessarily be the best method to evaluate PHLM instability, since mobility of the PHLM may be influenced by the force exerted with the probe as well as the degree of opening of the lateral tibiofemoral compartment [10]. The aspiration test allows to apply a standardized and evenly distributed traction force to the PHLM and thus reflects a reliable visual dynamic inspection of the lateral meniscus. The results of this study showed its superiority compared to the probing test and confirmed the improved detection of PHLM instability which was identified in 31% of ACL-injured patients. In the absence of the rare condition of an isolated PHLM instability (excluded in this study in the control group), the fact that none of the patients of the control group had a positive aspiration test allowed to literally rule out the possibility of false-positive evaluations and confirmed that the aspiration test is an effective method to evaluate PHLM instability in ACL-injured patients.

The pathophysiology of PHLM instability in ACL-injured knees remains insufficiently understood and its prevalence remains unknown. ACL injuries typically occur during a combined anterior translation and external rotation of the tibia against the femur [6] causing a blow of the posterolateral tibial plateau against the lateral femoral condyle typically resulting in a bone bruise or an impression fracture [9]. At the moment of anterior subluxation, both the posterolateral suspensory complex and the posterior root of the lateral meniscus are squeezed and massively strained between the femur and the tibia. The exerted shear forces may thus lead to the currently well-classified [6, 13] and easily identifiable PLMRT. The variability in the reported prevalence of type I–III PLMRT (7–17%) [5, 16, 17] and the high prevalence of lateral femoral and tibial bone bruises/impression fractures [24] in the area of the suspensory complex of the PHLM may suggest that a significant number of lesions to the PHLM are undiagnosed. Thus, a more subtle structural damage to the suspensory complex of the PHLM may occur in a significant amount of ACL-injured knees. These pathologic tissue alterations are either not macroscopically visible or they present with an elongation of the PLMRT, here classified as type IV lesions, the suggested extension of the Forkel classification. To the best of the authors’ knowledge, type 4 PLMRT or elongations of the posterior root of the lateral meniscus have not been described previously. This type of lesion corresponds to an incomplete tear of the root occurring at the time of injury where the meniscus tissue undergoes a severe distraction with an incomplete subsequent healing process (Fig. 1). In their respective PLMRT classifications, neither Laprade et al. [13] nor Forkel et al. [6] described this entity. In this study, this type of PLMRT was observed in 15 ACL-injured patients. Likewise, in ten additional patients, a PHLM instability without visible structural damage to the lateral meniscus could be identified and could represent an isolated damage to the suspensory complex of the PHLM.

The prevalence of type I, II, and III PLMRTs was 12% in the current series. This is in accordance with the previous publications where it was reported to be between 7 and 17% [5, 16, 17]. Adding the type IV to the other three types of PLMRT leads to a prevalence of 27% of PLMRT. In all but two patients (13/15; 87%) presenting with this specific type IV lesion, the aspiration test revealed a PHLM instability, whereas the probing test allowed to identify it in only 7% of patients. The poor agreement between the tests for this subpopulation supports the fact that the aspiration test is superior to the probing test in differentiating stable from unstable PHLM lesions that may need to be repaired. Further studies are needed to specifically investigate the biomechanical and clinical consequences of this type of lesion, such as their impact on dynamic rotatory laxity and the residual pivot-shift phenomenon after ACLR.

There are several limitations to the present study. Due to the multi-centric and international design of the study, and in order to be in accordance with ethical approval, no epidemiological data concerning the patients such as age, gender, and mechanism of injury could be analyzed. Following sample size calculation, the size of the ACL group cohort was limited to 103 patients. Larger cohorts or registries are needed to confirm the prevalence of PHLM instability in ACL-injured patients. Another limitation is that this study did not look into the correlation with MRI, which—at the current state of knowledge—seems unsuitable for the diagnosis of this pathology [1, 21]. It is also important to note that one of the main strengths of this study is the presence of a control group in which no instability of the PHLM could be demonstrated during both the probing and the aspiration tests. Further studies are needed to investigate the natural history, the biomechanical consequences and the surgical management of PHLM instabilities. Despite these limitations, the results of this study confirmed the superiority of the aspiration test over the probing test in the diagnostic of PHLM instability but also the high prevalence of this pathology in ACL-injured patients. The aspiration test should be used in routine clinical practice during systematic arthroscopic exploration of ACL-injured patients.

## Conclusion

Careful observation and examination of the PHLM with the aspiration test revealed a substantial amount of previously undiagnosed lateral meniscus instabilities in ACL-injured knees. The prevalence of PHLM instability as evaluated by the aspiration test was high (31%). The aspiration test was superior to the probing test in detecting an instability of the PHLM in a population of ACL-injured patients.
